# Correlation of Magnetic Resonance Imaging With the Histopathological Staging in Mid and Low Rectal Cancers

**DOI:** 10.7759/cureus.103886

**Published:** 2026-02-19

**Authors:** Kalla B Muralidhar, Joju Antony Sebastian, Raghu Ram Reddy

**Affiliations:** 1 Surgical Oncology, Mahatma Gandhi Cancer Hospital and Research Institute, Visakhapatnam, IND; 2 Surgical Oncology, Amala Institute of Medical Sciences, Thrissur, IND; 3 Surgical Oncology, Pi Health Cancer Hospital, Hyderabad, IND

**Keywords:** circumferential resection margin (crm), histopathology, magnetic resonance imaging(mri), neoadjuvant chemoradiotherapy, rectal cancer, tumor regression grade (trg)

## Abstract

Background

Magnetic resonance imaging (MRI) is the preferred modality for local staging of rectal cancer and plays a crucial role in guiding neoadjuvant therapy and surgical planning. However, its accuracy in post-neoadjuvant chemoradiotherapy (NACRT) restaging-particularly for tumor (T) stage, nodal status, and tumor regression-remains variable, largely due to post-treatment fibrosis, edema, and inflammatory changes that complicate differentiation between residual tumor and treatment-related effects.

Objectives

To evaluate the correlation between post-NACRT MRI staging and final histopathological staging in patients with carcinoma of the mid and low rectum, and to assess the accuracy of MRI in predicting tumor stage, nodal status, circumferential resection margin (CRM), and tumor regression grade.

Methods

This prospective observational study included 61 consecutive eligible patients with biopsy-proven, locally advanced, non-metastatic adenocarcinoma of the mid and low rectum treated between June 2020 and October 2021. All patients underwent baseline pelvic MRI for assessment of pre-treatment nodal status (cN0 and cN2), followed by long-course neoadjuvant chemoradiotherapy (NACRT) and repeat MRI six to eight weeks after treatment to document post-therapy nodal status. Post-NACRT MRI findings were compared with final histopathological staging, which served as the reference standard. Agreement was assessed using Cohen’s kappa statistic, and diagnostic accuracy was calculated.

Results

The median age of the study population was 48 years, with an almost equal gender distribution (males: 31/61, 50.8%; females: 30/61, 49.2%). Prior to neoadjuvant chemoradiotherapy (NACRT), most patients had locally advanced disease, with cT3-cT4 tumors in 57 patients (57/61, 93.4%). Pre-treatment nodal staging on MRI demonstrated cN2 disease in 35 patients (35/61, 57.4%) and cN0 status in the remaining patients.

Post-treatment MRI demonstrated significant downstaging, with cT3-cT4 disease observed in 34 patients (34/61, 55.7%). Following NACRT, nodal downstaging was evident, with cN0 status in 29 patients (29/61, 47.5%) and persistent cN2 disease in the remaining patients.

The overall accuracy of MRI for post-NACRT T staging was 54.1%, showing fair agreement with histopathology (κ = 0.37; p = 0.001), while nodal staging accuracy was higher at 73.8%, with moderate agreement (κ = 0.55; p = 0.001). Pathological complete response was observed in 14 patients (14/61, 23.0%), of whom MRI correctly identified three patients (3/14, 21.4%), yielding a sensitivity of 21.4% and a positive predictive value of 75.0%. MRI-based tumor regression grading demonstrated moderate agreement with pathological tumor regression grade (κ = 0.42), with an overall accuracy of 59.0%. MRI-guided post-treatment surgical planning resulted in negative circumferential resection margins in all patients.

Conclusion

High-resolution MRI is a valuable tool for post-NACRT assessment and surgical planning in mid and low rectal cancer. While MRI demonstrates moderate accuracy for nodal staging and tumor regression assessment, its performance in T-stage restaging remains limited due to post-treatment changes. MRI plays a critical role in achieving negative resection margins through individualized surgical planning. Advances in MRI technology and larger multicenter studies may further improve staging accuracy and clinical decision-making.

## Introduction

The incidence of rectal cancer is increasing globally, particularly in developing countries. According to Global Cancer Observatory (previously referred to as GLOBOCAN) 2020 data, colorectal cancer (CRC) is the third most commonly diagnosed cancer worldwide, while rectal cancer alone ranks as the eighth most common malignancy [1]. In 2020, India accounted for 28,260 new cases of rectal cancer out of a global total of 732,210 cases, with 16,149 deaths reported in India among 339,022 worldwide [1]. Comparison with GLOBOCAN 2018 data shows an increase of approximately 4,009 new rectal cancer cases annually in India, highlighting a significant rising trend [2].

Epidemiological studies have demonstrated a shift toward younger age groups being increasingly affected by rectal cancer worldwide [3]. This trend appears to be more pronounced in the Indian population, where a higher proportion of young patients are diagnosed compared with Western populations [4]. Similar observations among Indian immigrants in the United States suggest that a combination of genetic susceptibility, environmental exposure, and lifestyle factors may contribute to early-onset disease [5].

Prognosis in rectal cancer largely depends on the stage of disease at presentation. Diagnosis is usually established through digital rectal examination, endoscopic evaluation, and histopathological confirmation. However, these methods do not adequately assess the depth of tumor invasion or regional lymph node involvement, both of which are crucial prognostic indicators. As a result, modern rectal cancer management follows a multimodal approach, with treatment decisions made by a multidisciplinary team before surgery. These decisions are largely guided by preoperative staging using magnetic resonance imaging (MRI), which enables detailed assessment of mural and extramural tumor spread, nodal disease, and the circumferential resection margin (CRM).

Locally advanced disease, threatened or involved CRM, presence of extramural venous invasion (EMVI), and bulky nodal disease identified on MRI are established indications for neoadjuvant therapy. Neoadjuvant treatment may be delivered as short-course radiotherapy to reduce local recurrence or as long-course chemoradiotherapy to downstage tumors and improve resectability. Therefore, MRI plays a central role in the management of rectal cancer. At present, MRI best fulfills the requirements for preoperative local staging; however, accurate assessment of nodal status remains challenging.

Rectal cancer is a potentially curable disease, yet it carries a poor prognosis due to local recurrence and distant metastasis. Local recurrence is strongly associated with incomplete tumor resection [6,7] and is closely related to the distance between the tumor and the circumferential resection margin [8,9]. Total mesorectal excision (TME), which involves sharp dissection along the mesorectal fascia to remove the rectum and surrounding mesorectal fat, is widely accepted as the standard surgical technique [8]. The mesorectum consists of perirectal fat containing blood vessels, lymph nodes, and nerves enclosed within the mesorectal fascia [10]. The introduction of TME has significantly reduced local recurrence rates to below 10% in specialized centers, even without adjunctive therapy [8].

Neoadjuvant chemoradiotherapy is commonly employed in patients with extramural tumor spread or threatened CRM to improve local control and facilitate sphincter preservation. Multiple clinical trials have established preoperative chemoradiotherapy as standard treatment in patients at high risk of incomplete resection, including those with locally advanced tumors, extramural disease abutting the levator muscles, tumors close to the external anal sphincter, or lesions infiltrating the intersphincteric plane [11,12].

The role of imaging in rectal cancer has evolved significantly, with increasing emphasis on MRI-based preoperative assessment [13,14]. Advances in MRI technology, including the use of phased-array and endorectal coils, have greatly improved spatial resolution and staging accuracy [15,16]. Current evidence supports MRI as the most accurate modality for local staging of rectal cancer [17]. Furthermore, accurate preoperative MRI assessment of the relationship between the tumor and the circumferential resection margin has been shown to reduce the incidence of positive CRM and local recurrence, thereby improving oncologic outcomes [18].

The present study aims to evaluate the accuracy of MRI in predicting extramural tumor spread and lymph node involvement in rectal cancer and to compare MRI-based staging with final histopathological tumour, node, metastasis (TNM) staging.

## Materials and methods

This prospective observational study was conducted at the Departments of Surgical Oncology, Radiology, and Pathology, Basavatarakam Indo-American Cancer Hospital & Research Institute, Hyderabad, between June 2020 and October 2021. The study was approved by the Institutional Ethics Committee and was performed in accordance with the Declaration of Helsinki. Written informed consent was obtained from all participants.

Patients with biopsy-proven, locally advanced, non-metastatic adenocarcinoma of the mid and low rectum were prospectively enrolled. Eligibility required the lower margin of the tumor to be palpable on digital rectal examination and located within 6-7 cm from the anal verge. Patients planned for long-course neoadjuvant chemoradiotherapy were included. Exclusion criteria comprised distant metastasis or non-regional nodal disease, non-adenocarcinoma histology, tumors beyond 6-7 cm from the anal verge, patients undergoing primary surgery or short-course radiotherapy, medical unfitness for surgery, contraindications to MRI, prior pelvic radiotherapy or surgery, and refusal to consent.

All patients underwent baseline pelvic MRI followed by long-course neoadjuvant chemoradiotherapy. MRI examinations were performed using a GE SIGNA Explorer 1.5-Tesla scanner (GE Healthcare, Chicago, Illinois, USA) with a pelvic phased-array surface coil. To reduce motion artifacts, intravenous hyoscine butylbromide (20 mg) was administered immediately before imaging, and endorectal filling was routinely performed. The MRI protocol included high-resolution two-dimensional T2-weighted fast spin-echo sequences without fat suppression, acquired with a slice thickness of less than 4 mm in oblique axial (perpendicular to the tumor axis), sagittal, and oblique coronal planes. Diffusion-weighted imaging was obtained in all patients before and after neoadjuvant therapy. Additional large field-of-view axial T2-weighted images covering the pelvis from the aortic bifurcation to the anal sphincter were acquired to assess mesorectal, lateral pelvic, and distant lymph node chains.

Neoadjuvant chemoradiotherapy consisted of external beam radiotherapy delivered to a total dose of 50.4 Gy in 28 fractions (1.8 Gy per fraction), administered five days per week over six weeks using intensity-modulated radiotherapy (IMRT). Concurrent chemotherapy was given in the form of oral capecitabine at a dose of 825 mg/m² twice daily on radiotherapy days.

Post-treatment evaluation was performed six to eight weeks after completion of neoadjuvant therapy and included clinical examination and repeat pelvic MRI using the same imaging protocol. MRI assessments focused on post-treatment T stage, nodal status, circumferential resection margin (CRM), extramural venous invasion (EMVI), and tumor regression grade (mrTRG). Treatment response and surgical planning were discussed in a multidisciplinary tumor board, and the surgical approach was individualized based on post-treatment MRI findings, including tumor level, stage, nodal status, and involvement of adjacent structures.

All patients underwent definitive surgical resection. Histopathological examination of resected specimens was considered the reference standard. Tumors were staged according to the American Joint Committee on Cancer (AJCC) 8th edition TNM classification. Pathological tumor regression grading (pTRG) was assessed using the Mandard grading system, and circumferential resection margin status was documented.

Statistical analysis was performed using SPSS software version 25.0 (IBM Corporation, Armonk, New York, USA). Histopathological findings were used as the gold standard for comparison with post-neoadjuvant MRI findings. The diagnostic accuracy of MRI for T staging, N staging, and tumor regression grading was calculated using standard formulas. Agreement between MRI and histopathological staging was evaluated using Cohen’s kappa statistic. A p-value of less than 0.05 was considered statistically significant.

## Results

Patient demographics

A total of 61 patients with carcinoma of the mid and low rectum who received long-course neoadjuvant chemoradiotherapy followed by surgery were included in the study.

The median age of the study population was 48 years (range from 26-72 years). Most patients belonged to the 40-60-year age group (38/61; 62.3%), while 14 patients (22.9%) were younger than 40 years and nine patients (14.8%) were older than 60 years (Table 1).

**Table 1:** Baseline demographic characteristics of the study population (n = 61)

Variable	Category	n (%)
Age (years)	< 40	14 (22.9)
	40-60	38 (62.3)
	>60	9 (14.8)
Sex	Female	30(49)
	Male	31(51)
Body Mass Index (kg/m²)	Underweight(18.5 kg/m^2^)	6 (9.8)
	Normal (18.5-24.9 kg/m^2^)	35 (57.4)
	Overweight (25-29.9 kg/m^2^)	14 (23.0)
	Obese (30 kg/m^2^ and above)	6 (9.8)

**Figure 1:**
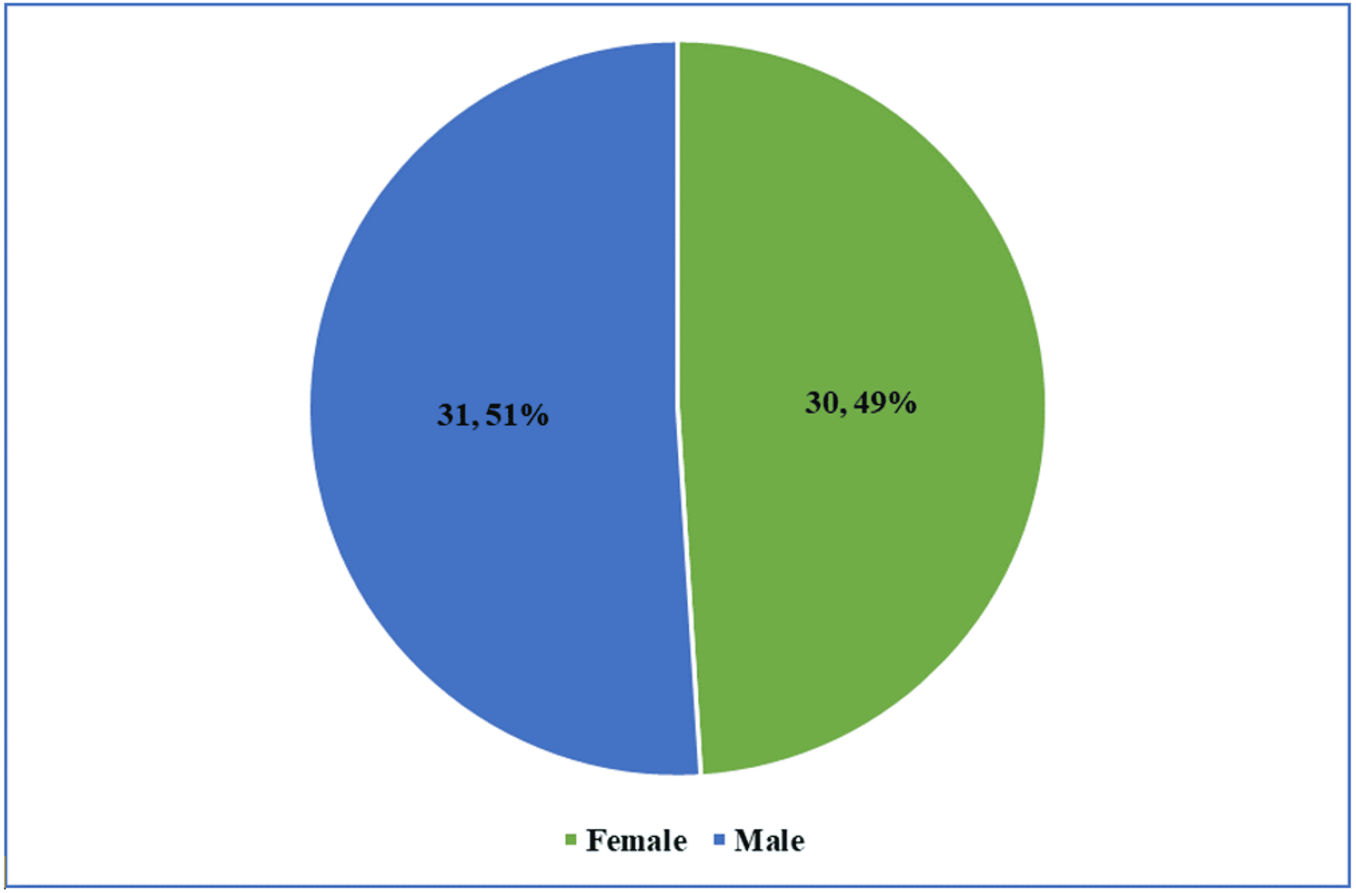
Gender distribution of the study population

There was an almost equal gender distribution, with 31 males (51%) and 30 females (49%) (Table 1, Figure 1).

**Figure 2:**
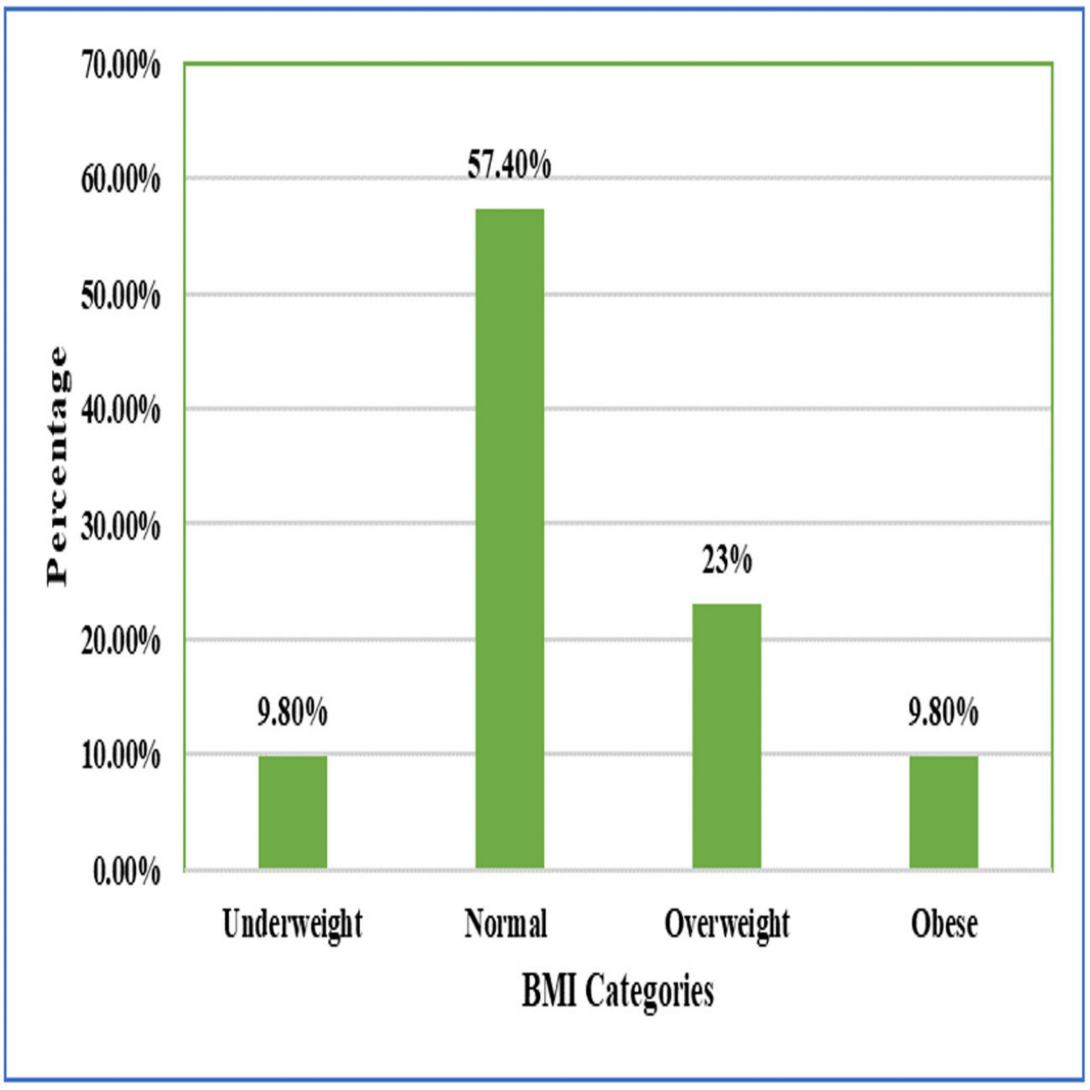
Distribution of body mass index (BMI) categories

The median body mass index (BMI) was 22.8 kg/m² (range from 16.4-34.7 kg/m²). The majority of patients were in the normal BMI category (35/61; 57.4%), followed by overweight (14/61; 23%), underweight (6/61; 9.8%), and obese (6/61; 9.8%) categories (Table 1, Figure 2).

Tumor characteristics

On initial clinical evaluation by digital rectal examination, the median distance of the lower margin of the tumor from the anal verge was 4 cm (range from 0-7 cm). On baseline pre-NACRT MRI, the median distance of the tumor from the anal verge was also 4 cm, with a wider range of 0-10 cm.

**Figure 3:**
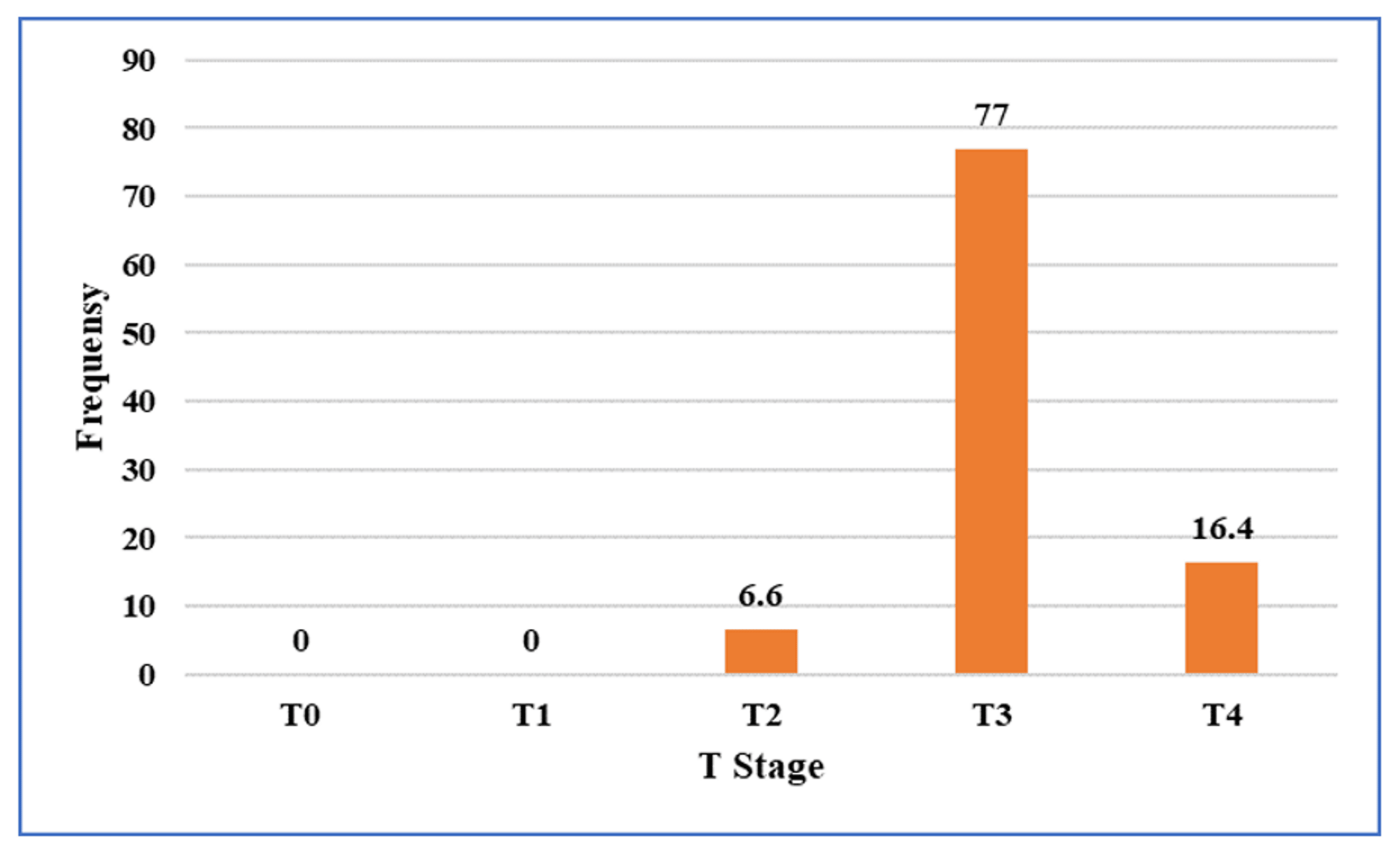
Distribution of tumor (T) stage assessed on pre-treatment pelvic magnetic resonance imaging

Based on pre-NACRT MRI findings, 41 patients (67.2%) had low rectal tumors, while 20 patients (32.8%) had mid-rectal tumors. Pre-NACRT MRI demonstrated that the majority of tumors were locally advanced. Nearly 94% (~57)of patients were staged as T3 or T4 on pre-NACRT MRI (Figure 3).

**Figure 4:**
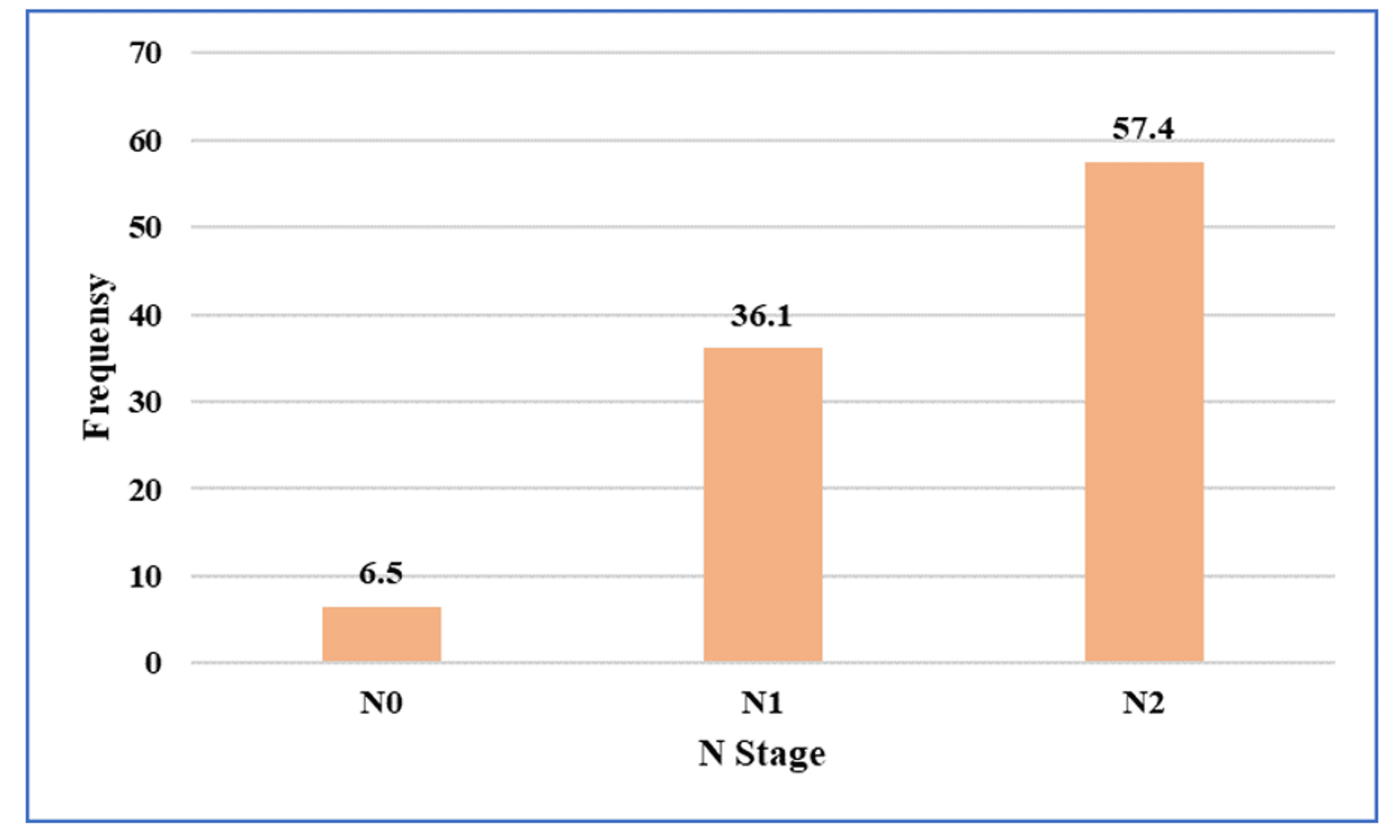
Distribution of nodal (N) stage assessed on pre-treatment pelvic magnetic resonance imaging

Regarding nodal status, 35 (57.4%) patients were staged as N2 on pre-NACRT MRI (Figure 4).

**Figure 5:**
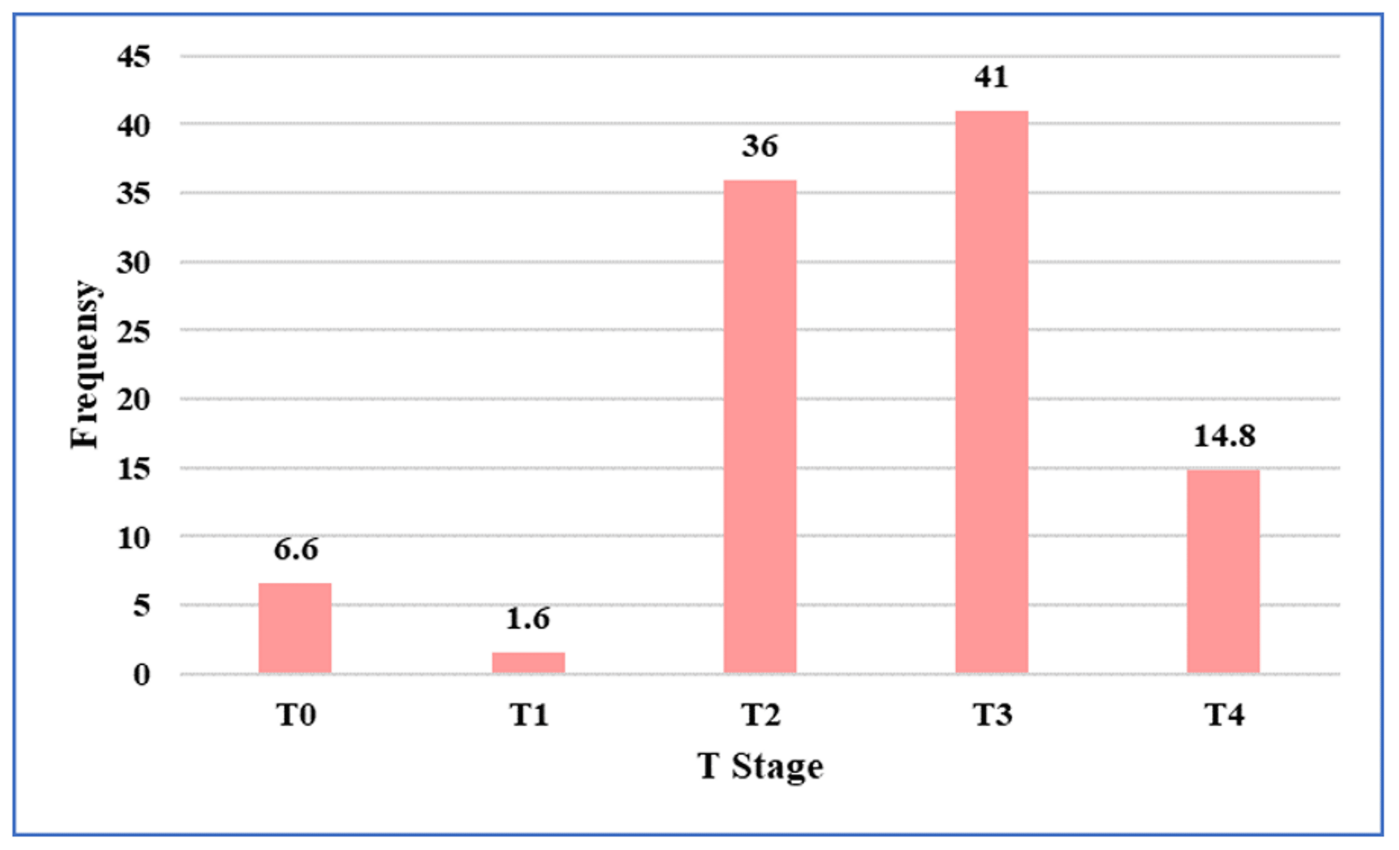
Distribution of tumor (T) stage assessed on post-treatment pelvic magnetic resonance imaging performed after completion of neoadjuvant chemoradiotherapy

Following neoadjuvant chemoradiotherapy, post-treatment MRI revealed evidence of tumor downstaging. T2 disease was observed in 22 (36%) of patients, while T3 disease was present in 25 (41%) on post-NACRT MRI (Figure 5).

**Figure 6:**
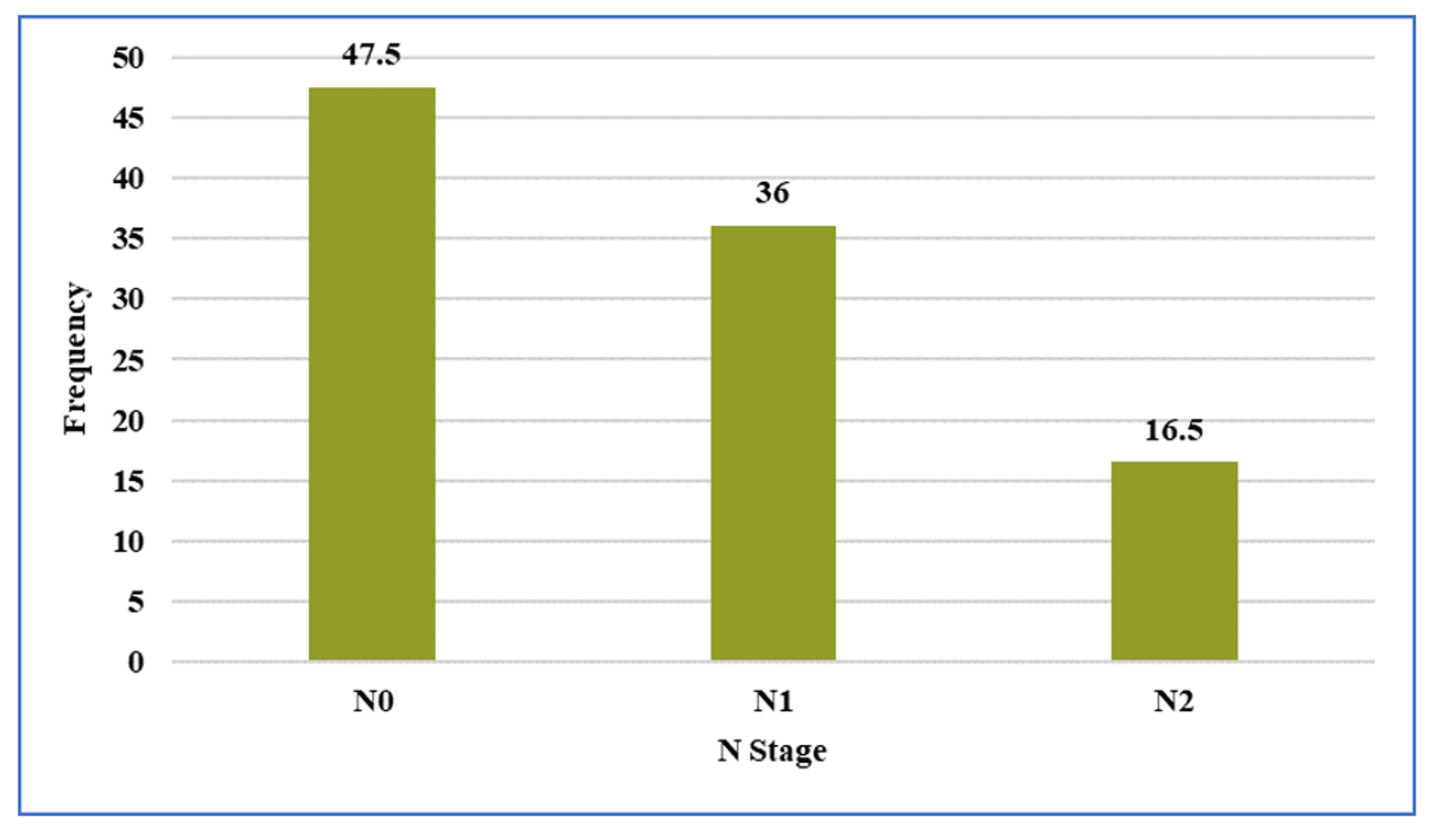
Distribution of nodal (N) stage assessed on post-treatment pelvic magnetic resonance imaging following neoadjuvant chemoradiotherapy

For nodal staging, 29 (47.5%) of patients were classified as N0 after NACRT (Figure 6).

Mesorectal fascia (MRF) and EMVI status

Assessment of MRF showed threatened or involved MRF in 18 patients on pre-NACRT MRI, which decreased to 14 patients on post-treatment MRI. Based on these MRI findings, individualized surgical planning was undertaken. Five patients underwent extralevator abdominoperineal excision (ELAPE) for levator ani involvement, and three patients underwent pelvic exenteration (one total and two posterior) due to invasion of adjacent pelvic organs identified on MRI. The remaining patients underwent abdominoperineal excision or low anterior resection according to tumor location. Importantly, all patients achieved negative circumferential resection margins (CRM) on final histopathological examination. This is due to an individualised surgical plan based on preoperative MRI. So the MRI has a great role in planning surgery to get a negative CRM.

Extramural venous invasion (EMVI) was detected in 40 patients on pre-NACRT MRI, which reduced to 14 patients on post-treatment MRI, indicating a favourable response to neoadjuvant therapy.

Surgical procedures

The types of surgical procedures performed following NACRT are detailed in Table 2. Abdominoperineal resection (APR) was the most commonly performed procedure (40 patients, 65.5%). Other procedures included low anterior resection (nine patients, 14.7%), ultra-low anterior resection (four patients, 6.6%), ELAPE (five patients, 8.2%), and pelvic exenteration (three patients, 5%). Partial vaginectomy was performed in five patients as part of APR, and pelvic lymph node dissection was carried out in three patients based on preoperative MRI findings.

**Table 2:** Distribution of surgical procedures performed following neoadjuvant chemoradiotherapy (NACRT)

Procedure performed	n (%)
Abdominoperineal resection (APR)	40 (65.5%)
Low anterior resection (LAR)	9 (14.7%)
Ultra low anterior resection (ULAR)	4 (6.6%)
Extralevator abdominoperineal excision (ELAPE)	5 (8.2%)
Pelvic exenteration	3 (5%)
Total	61

Correlation of post-NACRT MRI T Stage with pathological T stage

The correlation between post-NACRT MRI T staging and pathological T staging is summarized in Table 3. Pathological staging, considered the gold standard, demonstrated pT0 disease in 14 patients (23.0%), pT1 in four (6.6%), pT2 in 18 (29.5%), pT3 in 20 (32.8%), and pT4 in five patients (8.2%). Among 14 patients of pT0, one patient was upstaged as T1, 8 patients were upstaged as T2, and two patients were upstaged as T3 by preoperative MRI. The positive predictive value of MRI in identifying pCR was 75%, but the sensitivity was 21%.

**Table 3:** Correlation between post-NACRT MRI T stage and pathological T stage pT0 - pT4b = Post Neoadjuvant Chemoradiotherapy (NACRT) Pathological T staging (0-4b). mrT0 - mrT4b = Post Neoadjuvant Chemoradiotherapy (NACRT) MRI T staging (0-4b).

	pT0	pT1	pT2	pT3	pT4a	pT4b	Total
mrT0	3	1	0	0	0	0	4
mrT1	1	0	0	0	0	0	1
mrT2	8	2	11	1	0	0	22
mrT3	2	1	5	17	0	0	25
mrT4a	0	0	1	2	0	1	4
mrT4b	0	0	1	0	2	2	5
Total	14	4	18	20	2	3	61

Overall, MRI demonstrated an accuracy of 54.1% for post-NACRT T staging. Agreement between MRI-based T staging and pathological T staging, assessed using Cohen’s kappa statistics, was fair (κ = 0.37) and statistically significant (p = 0.001).

Correlation of post-NACRT MRI N stage with pathological N stage

The correlation between post-NACRT MRI nodal staging and pathological nodal staging is shown in Table 4. On histopathological examination, 44 patients (72.1%) had pN0 disease, eight patients (13.1%) had pN1 disease, and nine patients (14.8%) had pN2 disease. MRI accurately identified nodal status in the majority of cases, with an overall accuracy of 73.77%.

**Table 4:** Correlation between post neoadjuvant chemoradiotherapy (NACRT) MRI N stage and pathological N stage pN0 - pN2 = Post Neoadjuvant Chemoradiotherapy (NACRT) Pathological N staging (0-2). mrN0 - mrN2 = Post Neoadjuvant Chemoradiotherapy (NACRT) MRI N staging (0-2).

	pN0	pN1	pN2	Total
mrN0	29	0	0	29
mrN1	15	7	0	22
mrN2	0	1	9	10
Total	44	8	9	61

The level of agreement between MRI-based N staging and pathological N staging was moderate, with a kappa value of 0.55, which was statistically significant (p = 0.001). This moderate concordance underscores the clinical utility of post-NACRT MRI in guiding postoperative decision-making, particularly in tailoring the extent of surgical resection and planning adjuvant therapy.

Correlation of MRI tumor regression grade with pathological tumor regression grade

The comparison between MRI tumor regression grade (mrTRG) and pathological tumor regression grade (pTRG) is presented in Table 5. Pathological tumor regression was assessed using the Mandard grading system and served as the reference standard. MRI-based tumor regression grading showed moderate concordance with pathological TRG. Agreement between mrTRG and pTRG, assessed using Cohen’s kappa statistics, yielded a kappa value of 0.42, which was statistically significant (p = 0.001). The overall accuracy of MRI in assessing tumor regression following NACRT was 59%.

**Table 5:** Correlation between MRI tumor regression grade (mrTRG) and pathological tumor regression grade (pTRG) mr TRG1 - mr TRG5  = MRI tumor regression grade (mrTRG). pTRG1 - pTRG5  = Pathological tumor regression grade (pTRG).

	pTRG1	pTRG 2	pTRG 3	pTRG 4	pTRG 5	Total
mr TRG1	3	0	0	0	0	3
mr TRG2	7	6	1	0	0	14
mr TRG3	4	9	20	1	0	34
mr TRG4	0	1	1	6	0	8
mr TRG5	0	0	1	0	1	2
Total	14	16	23	7	1	61

## Discussion

The results of the present study demonstrate that high-resolution MRI remains a valuable adjunctive imaging modality for preoperative assessment and restaging of mid and low rectal cancer following long-course NACRT. MRI offers superior soft-tissue contrast, multiplanar imaging capability, and functional assessment, allowing detailed evaluation of the primary tumor, regional lymph nodes, and mesorectal fascia. However, the correlation between post-NACRT MRI findings and pathological staging in this study was moderate, particularly for tumor (T) staging, reflecting the known challenges posed by post-treatment fibrosis, edema, and inflammatory changes. In routine clinical practice, MRI demonstrates reasonable,but not definitive,accuracy in documenting tumor regression and nodal downstaging after chemoradiotherapy and should therefore be regarded as a complementary tool that informs surgical planning rather than a substitute for histopathological assessment.

Patient selection criteria for long-course chemoradiotherapy vary across institutions. At our center, patients with locally advanced rectal cancers involving the mid and lower rectum were selected for NACRT based on MRI findings and clinical assessment. These included tumors with threatened or involved circumferential resection margins, extramural tumor invasion, nodal disease, tumors close to the external anal sphincter, and lesions with suspected intersphincteric involvement. Treatment decisions were made through a multidisciplinary team approach, emphasizing the central role of MRI in guiding individualized treatment strategies.

Preoperative long-course chemoradiotherapy followed by surgery has been shown to improve sphincter preservation, reduce local recurrence rates, and improve overall survival. In patients achieving complete clinical response, a “watch-and-wait” approach is increasingly being explored, although its oncological safety remains controversial. Therefore, accurate post-treatment restaging is crucial to guide optimal individualized management decisions in rectal cancer.

MRI assessment after NACRT is technically challenging because of its limited ability to reliably differentiate residual viable tumor from post-treatment fibrosis, rectal wall thickening, and inflammatory infiltration. These factors may lead to overestimation or underestimation of tumor extent. A systematic review and meta-analysis reported a mean sensitivity of 50.4% for MRI in post-CRT T restaging [19]. Other studies have similarly demonstrated suboptimal accuracy, with reported T-stage accuracy of 47-52% and nodal staging accuracy of 64-68% using MRI [20].

In the present study, 93% of patients were staged as cT3 or cT4 before NACRT, which reduced to 56% following chemoradiotherapy, indicating a reasonable downstaging response. The overall accuracy of MRI in post-treatment T staging was 54.1%, with fair agreement between MRI and pathological staging (κ = 0.37). These findings are comparable to those reported by Shufang Zhan et al., who demonstrated an overall accuracy of 49% for MRI restaging after CRT [21]. However, higher accuracies have been reported by Liping Xu et al. (79.6%) and Jong Hoon Lee et al. (64.7%) [22,23]. The improved performance in these studies may be attributed to the use of 3-Tesla MRI scanners, which provide higher spatial resolution and better signal-to-noise ratio.

In our study, MRI demonstrated limited sensitivity (21%) in detecting pathological complete response (pCR), although the positive predictive value was relatively high (75%). The reduced sensitivity was primarily due to overstaging of pT0 tumors, as post-radiation fibrosis and inflammatory changes often mimic residual disease on MRI. This highlights the need for specialized expertise in MRI interpretation for rectal cancer. The incorporation of diffusion-weighted imaging may improve differentiation between fibrosis and residual tumor. Furthermore, the use of 3-Tesla MRI may reduce overstaging due to improved rectal wall visualization compared with 1.5-Tesla systems [24].

Lymph node involvement remains an independent prognostic factor in rectal cancer outcomes. Accurate nodal assessment is challenging, as no single imaging modality or universally accepted criteria reliably predict nodal metastasis. In our study, 94% of patients were staged as cN1 or cN2 before NACRT, while post-treatment staging showed a shift toward cN0 and cN1 disease in 83.5% of patients, reflecting effective nodal downstaging. MRI demonstrated an overall nodal staging accuracy of 73.77%, with moderate agreement between MRI and histopathology (κ = 0.55). The improved nodal staging performance in our study may be attributed to the use of combined morphological criteria, including irregular nodal borders, heterogeneous signal intensity, round shape, and size greater than 5 mm.

Evaluation of the mesorectal fascia (MRF) is one of the most clinically significant strengths of MRI. In the present study, post-NACRT MRI identified threatened or involved MRF in 14 patients, allowing tailored surgical approaches such as ELAPE and pelvic exenteration when indicated. Importantly, all patients achieved negative circumferential resection margins on final histopathology, underscoring the role of MRI-guided individualized surgical planning. These findings are consistent with the Low Rectal Cancer Study (short title: MERCURY II) study, which reported a positive CRM rate of only 9% with MRI-based surgical planning [25].

Tumor regression grading (TRG) is increasingly used to assess response to neoadjuvant treatment. In our study, MRI-based TRG demonstrated moderate agreement with pathological TRG (κ = 0.42), with an accuracy of 59%. Francesco Sclafani et al. reported only fair agreement between mrTRG and pTRG, concluding that mrTRG cannot reliably serve as a surrogate for pathological assessment [26]. While mrTRG provides useful non-invasive information regarding treatment response, it should be interpreted in conjunction with other imaging parameters and clinical findings. Further studies are required to establish its role in identifying complete responders and guiding non-operative management strategies.

Limitations of the study

This study has certain limitations that should be considered while interpreting the results. Being a single-centre study with a modest sample size, the findings may have limited external validity. Post-neoadjuvant MRI assessment is inherently challenging because treatment-related fibrosis and inflammatory changes can obscure residual disease, potentially affecting staging accuracy, particularly for T staging and tumor regression assessment. In addition, the use of a 1.5-Tesla MRI system and the absence of long-term oncological outcome analysis may have influenced the precision and clinical extrapolation of the results. Nevertheless, the prospective design, uniform imaging protocol, and correlation with histopathology provide meaningful insights into the role of MRI in post-NACRT assessment of rectal cancer, and the findings lay the groundwork for future larger, multicentric studies with advanced imaging techniques and extended follow-up.

## Conclusions

This prospective observational study demonstrates that magnetic resonance imaging plays a pivotal role in the post-neoadjuvant chemoradiotherapy assessment of patients with stage II and III mid and low rectal cancer. MRI provided valuable information regarding tumor response, nodal status, mesorectal fascia involvement, and resection planes, thereby facilitating individualized, multidisciplinary surgical planning. A meaningful degree of tumor and nodal downstaging following neoadjuvant treatment was observed, reinforcing the effectiveness of long-course chemoradiotherapy in locally advanced rectal cancer. While MRI showed variable performance in accurately predicting post-treatment T stage, it proved more reliable in nodal assessment and in evaluating surgical margins.

Importantly, MRI-based assessment of the mesorectal fascia enabled tailored surgical approaches and contributed to the achievement of negative circumferential resection margins in all patients, underscoring its clinical relevance in operative planning. MRI-based tumor regression grading demonstrated moderate concordance with pathological assessment and may serve as a useful adjunct in evaluating treatment response, although it cannot replace histopathology. Overall, high-resolution MRI remains integral to post-treatment evaluation, surgical decision-making, and optimization of local disease control in rectal cancer. Future large-scale, multicenter studies using advanced imaging techniques are warranted to further refine the role of MRI in post-neoadjuvant staging and response assessment.
